# Autosomal recessive congenital ichthyosis caused by a novel variant in cornifelin gene: A case report

**DOI:** 10.1016/j.jdcr.2024.07.004

**Published:** 2024-07-23

**Authors:** Basel Almalki, Yara Alghamdi, Abdullah Aman, Samer Alamri, Alhussain Alshareef, Ali Alraddadi

**Affiliations:** a Dermatology Department, King Fahad General Hospital, Jeddah, Saudi Arabia; b Department of Dermatology, King Abdullah Medical Complex, Jeddah, Saudi Arabia; c Dermatology Department, King Abdulaziz Medical City, Ministry of National Guard, Jeddah, Saudi Arabia

**Keywords:** ARCI, autosomal recessive congenital ichthyosis, CNFN gene, gene mutation, ichthyosis

## Introduction

Ichthyoses are genetically determined monogenic (Mendelian) disorders of cornification in which abnormal differentiation and desquamation of the epidermis result in a defective cutaneous barrier.^1^, ^2^ It represents a clinically and etiologically heterogeneous group of conditions. The most common forms of ichthyosis are autosomal dominant ichthyosis vulgaris and X-linked recessive steroid sulfatase-related ichthyosis, both of which are nonsyndromic ichthyoses.^3^ Nonsyndromic ichthyoses are limited to skin symptoms, whereas syndromic forms are classified according to the additional symptoms. In populations with a high rate of consanguinity; however, autosomal recessive forms represent a substantial fraction of congenital ichthyosis with an extensive list of genes that underlie both syndromic and nonsyndromic subtypes.^1^

The cornified envelope (CE) is a crucial structure for epidermal barrier function. The terminal differentiation of keratinocytes concludes with the substitution of the plasma membrane by the CE, composed of several covalently cross-linked proteins. Lipids, forming the cornified lipid envelope, cover the CE. Once keratinocytes assemble the CE/cornified lipid envelope and lose their nucleus and cytoplasmic organelles, they are known as corneocytes. These cell remnants comprise the stratum corneum, the major contributor to the body’s surface cover and the cutaneous water barrier. Pathogenic variants of genes responsible for various components of the CE can result in ichthyosis, as seen in both ichthyosis vulgaris and lamellar ichthyosis. In ichthyosis vulgaris, for instance, abnormalities in filaggrin can lead to this condition. Filaggrin facilitates the formation of tightly packed squamous cells by cross-linking the keratins in the CE. Additionally, filaggrin's degradation into moisture-retaining amino acids contributes to skin hydration. Consequently, a deficiency in filaggrin results in compromised keratinization and xerosis. In LI, conversely, the function of Transglutaminase-1, crucial for linking proteins within the CE, is impaired due to pathogenic variants. This disruption in protein linkage leads to the development of pathological ichthyosis.^3^, ^4^, ^5^

Cornifelin (CNFN) has recently been identified as a component of the CE in human skin. This paper presents 2 cases of brothers with autosomal recessive congenital ichthyosis who were found to possess a novel variant in the CNFN gene.

## Case presentation

**Fig 1:**
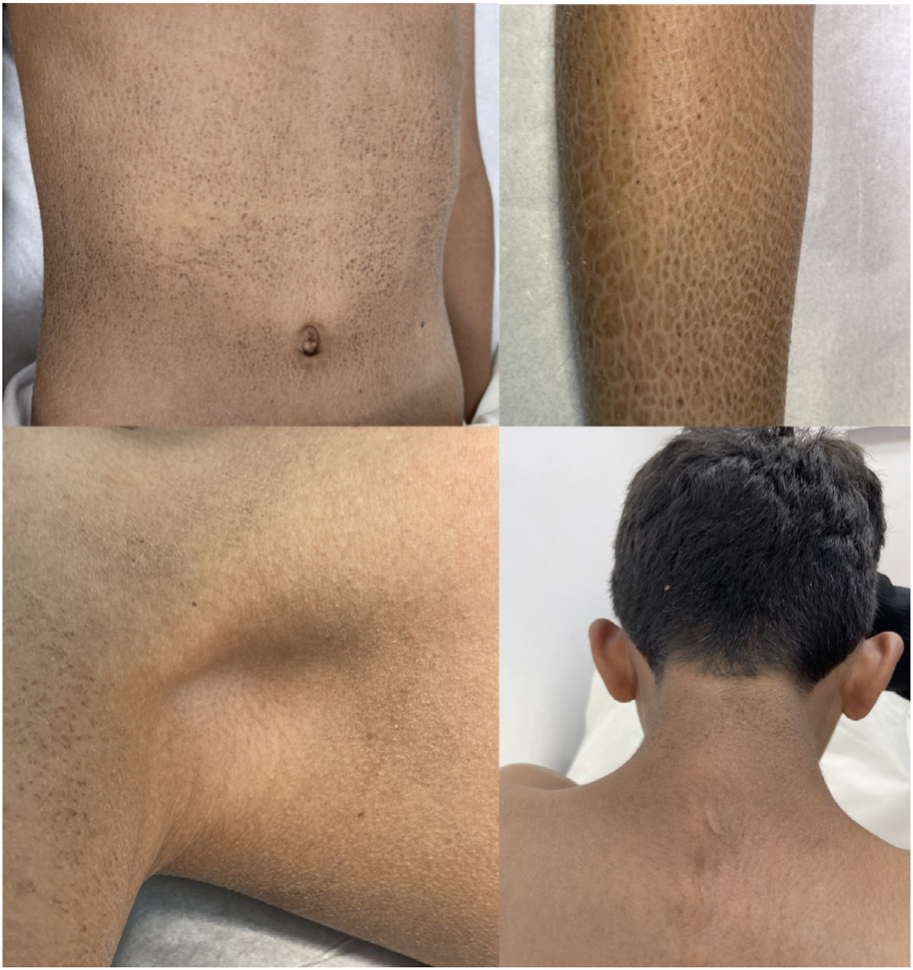
Diffuse dry, *brown*, adherent scales with a polygonal ichthyotic appearance.

**Fig 2:**
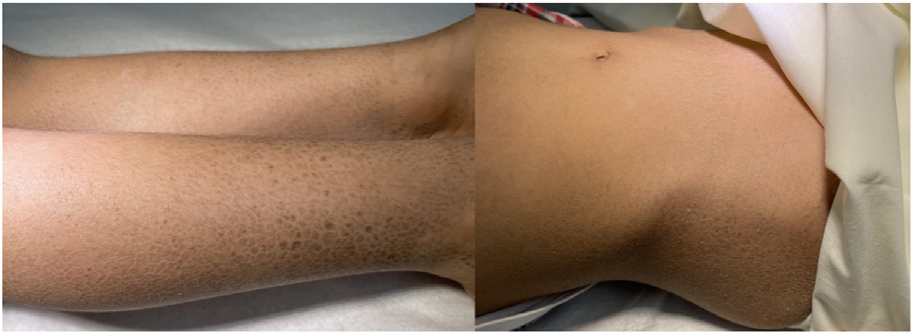
Diffuse dry, *brown*, adherent scales with a polygonal ichthyotic appearance.

Two brothers suffering from ichthyosis have been under the care of a dermatology department since birth. Their parents are consanguineous, and the boys are now 12 (case 1) and 5 (case 2) years old. Both were born full-term via spontaneous vaginal deliveries with no complications before, during, or after birth. Neither child developed colloidal membranes. From birth, the boys exhibited skin lesions characterized by polygonal brown, plate-like scales covering their bodies, except for their faces, palms, soles, and flexural areas (Figs 1 and 2). There were no abnormalities in their hair, nails, or mucous membranes and no signs of palmoplantar keratoderma.

Extracutaneous examinations – including ophthalmologic, pulmonary, and cardiac – did not reveal any abnormalities in either patient. Case 1 has healthy teeth but was noted to have retractile testes. (Fig 1). An ultrasound showed that both testicles were in the scrotal sac but had moved with stimulation to the distal parts of the inguinal canals, indicating bilateral retractile testes. Despite this, the testes appeared normal in shape and size with maintained vascularity, as shown in the sonographic assessment. Case 2 has had several dental appointments for tooth decay over recent years (Fig 2). Apart from these, both patients exhibit normal growth patterns and no other medical abnormalities.

**Fig 3:**
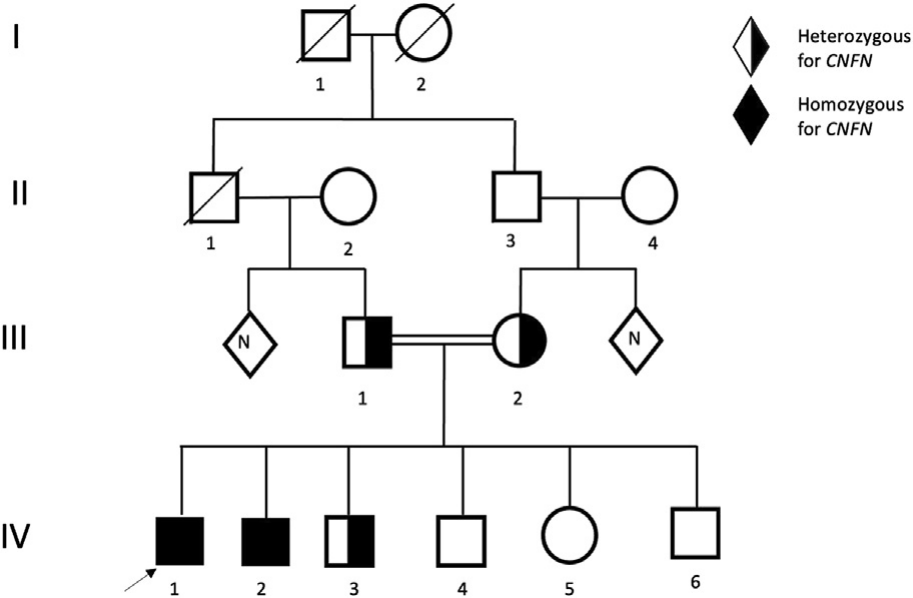
Family pedigree.

Genetic testing of both patients conducted through Whole Exome Sequencing identified that they carry the same homozygous splice site variant in CNFN: (NM_032488.4):c.113-1G > Tp.?(chr19:42891629; hg19). Patients have 4 unaffected siblings, 3 brothers and 1 sister. Carrier status analysis showed that both unaffected parents and one unaffected brother are heterozygous carriers of the same CNFN variant. Both patients are presently under regular follow-up, involving routine skin and extracutaneous examinations (Fig 3). The patients’ condition is managed with regular applications of moisturizing creams and keratolytic agents, such as urea creams and petroleum oil.

Skin conditions for both patients have been stable over time. No new skin, hair, nails, or mucosal lesions. No signs of cutaneous or mucosal infections. Both cases report no heat or cold intolerance. Both brothers are up to their age and attend school regularly.

## Discussion

The CNFN gene, situated on chromosome 19q13, consists of 4 exons and 3 introns, encoding a 112-amino acid protein with a mass of 12.4 kDa.^6^ Studies suggest that the CNFN protein supports the formation of the skin’s CE. Immunoprecipitation techniques, implemented in a transgenic mouse model with overexpressed human CNFN, have demonstrated an association between CNFN protein and either involucrin or loricrin, major components of the CE. Its expression is notably increased in psoriatic skin, atopic dermatitis, and mycosis fungoides by 18.5-fold, 14.3-fold, and 4.6-fold, respectively. Despite the upregulation of CNFN expression in psoriatic skin in vivo, a transgenic mouse model overexpressing human CNFN did not exhibit abnormal epidermal differentiation or manifest psoriatic lesions. However, this mouse model did display decreased expression of loricrin and upregulation of involucrin, which are alterations typically associated with psoriasis.^6^ The CNF gene is expressed in the uterus, but no data are reporting any phenotype in females. CNFN is also expressed in the mucosal tissue; however, patients in our cases have no mucosal abnormalities.

In a skin model where CNFN was reduced, hyperkeratosis was observed, along with a decrease in keratohyalin granules on hematoxylin and eosin stain. These findings may clinically correlate to the skin manifestations seen in our case study.^7^

Although there is no mouse knockout model of CNFN, mice deficient in Zdhhc13 enzyme were found to have undetectable cornifelin level in the skin. Zdhhc 13 is a Palmitoylation enzyme that uses CNFN as a specific substrate.^8^ These mice were found to have skin abnormalities that share some similarities with our cases like hyperkeratosis and disturbed skin barrier.

To date, pathogenic variants in the CNFN gene have not been associated with a specific phenotype or disease in the Online Mendelian Inheritance in Man database. In our cases, individuals were identified as carrying a novel homozygous splice site variant in CNFN: (NM_032488.4):c.113-1G > Tp.? (chr19:42891629; hg19). This variant has not been previously reported in the literature (HGMD 2019.4), and according to the genomAD database, no allele frequencies in the general population have been documented. The variant is classified as likely pathogenic based on American College of Medical Genetics and Genomics criteria in varsome database and assigned PVS1 (pathogenic, very strong) as it is a splice site variant that undergo nonsense-mediated RNA decay. The variant results in exon skipping, disrupting the reading frame, and removing 40.4% of the transcript. These findings demonstrate the pathogenicity of the variant and the harmful effects on the gene product. In silico predictions suggest that this CNFN variant has a deleterious effect on the gene (Aggregated Prediction score = 0.8, SpliceAI = 0.97, dbscSNV Ada = 1).^9^

In conclusion, we detail the cases of 2 brothers with autosomal recessive congenital ichthyosis associated with and likely resulting from a novel pathogenic variant in the CNFN gene. This case underscores the significance of the CNFN protein in forming the cornified cell envelope.

## Conflicts of interest

None disclosed.
